# Progressive logopenic/phonological aphasia: Erosion of the language network

**DOI:** 10.1016/j.neuroimage.2009.08.002

**Published:** 2010-01-01

**Authors:** Jonathan D. Rohrer, Gerard R. Ridgway, Sebastian J. Crutch, Julia Hailstone, Johanna C. Goll, Matthew J. Clarkson, Simon Mead, Jonathan Beck, Cath Mummery, Sebastien Ourselin, Elizabeth K. Warrington, Martin N. Rossor, Jason D. Warren

**Affiliations:** a Dementia Research Centre, Institute of Neurology, Queen Square, London WC1N 3BG, UK; b MRC Prion Unit, Department of Neurodegenerative Disease, UCL Institute of Neurology, University College London, Queen Square, London, WC1N 3BG, UK; c Centre for Medical Image Computing, University College London, Gower Street, London, WC1E 6BT, UK

**Keywords:** Primary progressive aphasia, Frontotemporal dementia, Frontotemporal lobar degeneration, Logopenic aphasia

## Abstract

The primary progressive aphasias (PPA) are paradigmatic disorders of language network breakdown associated with focal degeneration of the left cerebral hemisphere. Here we addressed brain correlates of PPA in a detailed neuroanatomical analysis of the third canonical syndrome of PPA, logopenic/phonological aphasia (LPA), in relation to the more widely studied clinico-anatomical syndromes of semantic dementia (SD) and progressive nonfluent aphasia (PNFA). 32 PPA patients (9 SD, 14 PNFA, 9 LPA) and 18 cognitively normal controls had volumetric brain MRI with regional volumetry, cortical thickness, grey and white matter voxel-based morphometry analyses. Five of nine patients with LPA had cerebrospinal fluid biomarkers consistent with Alzheimer (AD) pathology (AD-PPA) and 2/9 patients had progranulin (*GRN*) mutations (*GRN*-PPA). The LPA group had tissue loss in a widespread left hemisphere network. Compared with PNFA and SD, the LPA group had more extensive involvement of grey matter in posterior temporal and parietal cortices and long association white matter tracts. Overlapping but distinct networks were involved in the AD-PPA and *GRN-*PPA subgroups, with more anterior temporal lobe involvement in *GRN*-PPA. The importance of these findings is threefold: firstly, the clinico-anatomical entity of LPA has a profile of brain damage that is complementary to the network-based disorders of SD and PNFA; secondly, the core phonological processing deficit in LPA is likely to arise from temporo-parietal junction damage but disease spread occurs through the dorsal language network (and in *GRN-*PPA*,* also the ventral language network); and finally, *GRN* mutations provide a specific molecular substrate for language network dysfunction.

## Introduction

Recent research in clinical aphasiology has seen increasing attention played to the role of distributed networks in language dysfunction (Hillis, 2007; Rohrer et al., 2008a). The paradigmatic disorders that illustrate this concept are the primary progressive aphasias (PPA): a group of neurodegenerative syndromes that affect predominantly left hemispheric language networks. These disorders were initially described within the non-Alzheimer dementia spectrum (Mesulam, 2001, 2003) and have been incorporated into consensus criteria for frontotemporal lobar degeneration (FTLD) (Neary et al., 1998). Although two canonical subtypes were originally described—semantic dementia (SD) and progressive non-fluent aphasia (PNFA)—more recent work has attempted to refine the classification of PPA with several papers describing a third subtype, known as the logopenic/phonological variant of PPA (LPA) (Gorno-Tempini et al., 2004, 2008). Whereas the brain correlates of SD and PNFA have been widely studied less information is available for the LPA subtype. Although initially reported in early descriptions of PPA (Mesulam, 1982, 2001; Kertesz et al., 2003), LPA was first described in detail by Gorno-Tempini et al. (2004) and then expanded upon by the same group in a series of follow-up studies (Rosen et al., 2006; Amici et al., 2006; Gorno-Tempini et al., 2008; Rabinovici et al., 2008; Brambati et al., 2009; Wilson et al., 2009). The disorder has been characterized as a primary phonological loop deficit resulting in impaired verbal short term (phonological) memory, impaired sentence repetition and comprehension with sparse spontaneous speech and frequent prolonged word-finding pauses. Anatomically, brain atrophy accompanying LPA has a perisylvian distribution that overlaps with PNFA, however a left temporo-parietal correlate has been emphasized in group structural and metabolic neuroimaging studies (Gorno-Tempini et al., 2008; Rabinovici et al., 2008; Wilson et al., 2009). Post-mortem and amyloid imaging studies have emphasized the association of LPA with Alzheimer's disease (AD) pathology (Mesulam et al., 2008; Rabinovici et al., 2008). A parallel focus in the dementia literature has been atypical language variants of AD (Galton et al., 2000; Croot et al., 2000; Alladi et al., 2007; Stopford et al., 2007, 2008), although reports have often been based retrospectively on post-mortem data (Galton et al., 2000; Croot et al., 2000; Alladi et al., 2007). The phenotype described in many of these cases is similar to the LPA syndrome though correlation is not straightforward as AD pathology has also been associated (albeit less commonly) with other PPA phenotypes (Knibb et al., 2006; Gerstner et al., 2007; Rabinovici et al., 2008; Pereira et al., 2009). PPA may be familial and it has recently been shown that some of these patients have mutations in the progranulin (*GRN*) gene (Snowden et al., 2006; Mesulam et al., 2007). The language phenotype of patients with GRN mutations has been little studied although they have been described as nonfluent with a prominent anomia (Snowden et al., 2006, 2007; Rohrer et al., 2008c).

The brain correlates of LPA are of considerable neurobiological as well as clinical interest. Preliminary studies suggest predominant left temporo-parietal involvement in this disorder, implying a pattern of distributed brain damage that is complementary to SD and PNFA (Gorno-Tempini et al., 2004, 2008; Wilson et al., 2009). This study was designed to identify brain imaging features of LPA in relation to SD and PNFA using complementary imaging techniques of volumetric measures, cortical thickness analysis and voxel-based morphometry in a consecutive series of patients presenting with PPA.

## Materials and methods

### Subject characteristics

Thirty-three consecutive patients fulfilling a diagnosis of PPA according to current criteria (Mesulam, 2001, 2003) and not fulfilling criteria for an alternative dementia syndrome were recruited from the tertiary Specialist Cognitive Disorders Clinic of the National Hospital of Neurology and Neurosurgery, London, UK. All patients had a structured clinical history, neurological examination and screening cognitive assessment (Warrington, 2003) performed by an experienced cognitive neurologist (JW, MNR, CM). Based on this initial assessment and independent of brain imaging findings, we assigned patients to three syndromic groups: 9 (27%) were categorized as SD based on the presence of fluent speech, anomia, impaired word comprehension and deficits in non-verbal semantic domains (modified Neary criteria as per Adlam et al., 2006; Neary et al., 1998; Adlam et al., 2006); 14 (42%) were categorized as PNFA based on the presence of apraxia of speech and/or agrammatism and relatively intact single word comprehension (modified Neary criteria as per Gorno-Tempini et al., 2004; Neary et al., 1998; Gorno-Tempini et al., 2004); and 10 patients (30%) were categorized as LPA based on the presence of word-finding pauses in spontaneous speech (in the absence of a motor speech deficit), impaired repetition and comprehension of sentences and poor verbal short-term memory (Gorno-Tempini et al., 2004, 2008). One patient in the LPA group had a cardiac pacemaker in situ and therefore only 9 patients were included in this study. A control group of 18 cognitively normal healthy subjects matched for gender and age was also included. Research ethics approval for this study was obtained from the National Hospital for Neurology and Neurosurgery and University College London Hospitals Research Ethics Committees.

All patients were screened for mutations in the *MAPT* (exons 1 and 9–13), *GRN* (all exons) and *VCP* (exons 3, 5, 6 and 10) genes. Two *GRN* mutations were found in patients who had received the diagnosis of LPA: C31fs, 603_603insC (both previously described in Beck et al., 2008); a third mutation (R493X) was found in the LPA patient excluded from the study. No mutations were found in other genes screened. Of note, total-tau and Aβ42 cerebrospinal fluid (CSF) biomarker data were available for six of the seven LPA cases without *GRN* mutations: all had raised levels of tau although only five of the six patients also had low Aβ42, a CSF profile previously described in association with pathologically proven Alzheimer's disease (Sunderland et al., 2003).

Demographic data, Mini-Mental State Examination (MMSE, Folstein et al., 1975), Frontal Assessment Battery (FAB, Dubois et al., 2000), Clinical Dementia Rating (CDR, Morris, 1993) in each of the four groups are presented in Table 1. The general neurological examination was normal in the majority of patients. However, three patients in the PNFA group had a parkinsonian syndrome: one had features of a corticobasal syndrome and two had features of a progressive supranuclear palsy syndrome. Formal neuropsychological assessment was performed in all of the subjects (Table 1, details of tests available in supplementary information) with characteristic neuropsychological profiles exhibited by both the SD group (anomia, single word comprehension, intact repetition and surface dyslexia) and the PNFA group (impaired sentence comprehension, poor single word and sentence repetition with apraxic errors, mild anomia and phonological dyslexia). In comparison, patients with LPA exhibited more severe verbal short term memory deficits (digit span forwards), sentence comprehension and sentence repetition deficits than the other groups, similar to previous descriptions (Gorno-Tempini et al., 2004, 2008). On a single-word comprehension (word–picture matching) task, patients with LPA performed worse than the PNFA group but better than the SD group. Other features in the LPA group included deep/phonological dyslexia (difficulty reading non-words, but with a mixture of error types, including semantic and visual errors: Coltheart, 1980; Crisp and Lambon Ralph, 2006; Brambati et al., 2009) and other left parietal lobe deficits (impaired limb praxis, dyscalculia), also consistent with previous reports of LPA (Amici et al., 2006; Gorno-Tempini et al., 2008).

Patients receiving a diagnosis of LPA with GRN mutations (GRN-PPA) were compared clinically and neuropsychologically with LPA patients who had CSF biomarkers consistent with AD (AD-PPA): subgroup data are summarized in Table 1. The AD-PPA group undoubtedly fit the proposed criteria for LPA with impoverished spontaneous speech, a phonological store deficit (decreased forwards digit span) and impairment of both sentence repetition and comprehension (Gorno-Tempini et al., 2008). In comparison the *GRN-*PPA group exhibit similar features but with significantly worse (*p* = 0.02) performance on reading irregular words and a trend to more severe impairments of naming, single word comprehension and repetition.

### Brain image acquisition and analyses

MR brain images were acquired on a 1.5-T GE Signa scanner (General Electric, Milwaukee, WI) using an IR-prepared fast SPGR sequence (TE = 5 ms, TR = 12 ms, TI = 650 ms). T1-weighted volumetric images were obtained with a 24-cm field of view and 256 × 256 matrix to provide 124 contiguous 1.5-mm-thick slices in the coronal plane.

#### Volumetric imaging

Image analysis was performed using the MIDAS software package (Freeborough et al., 1997). Scans were outlined using a rapid semi-automated technique which involves interactive selection of thresholds, followed by a series of erosions and dilations. This yields a region which separates brain from surrounding CSF, skull and dura. Scans and associated brain regions were subsequently transformed into standard space by registration to the Montreal Neurological Institute (MNI) Template (Mazziotta et al., 1995). The left and right hemispheric regions were defined using the MNI average brain which was split by dividing the whole volume along a line coincident with the interhemispheric fissure. An intersection of each individual's brain region and the hemispheric regions defined on the MNI template was generated to provide a measure of brain volume in left and right hemispheres (Chan et al., 2001; Beck et al., 2008). Left/right volume ratios were subsequently calculated. Volumetric analysis of specific subcortical structures (hippocampus, amygdala, caudate and brainstem) was performed using the Freesurfer image analysis suite version 4.0.3 (http://surfer.nmr.mgh.harvard.edu/) (Fischl et al., 2002), on a 64-bit Linux CentOS 4 Sun Grid Engine Cluster. Initially, the four groups (SD, PNFA, LPA and controls) were compared statistically by looking at the two-tailed contrasts between the group means using a linear regression model (STATA8^©^, Stata Corp, College Station, TX). Subgroup analyses with SD, PNFA, *GRN*-PPA only, AD-PPA only and controls were also performed.

#### Cortical thickness analysis

Cortical reconstruction and thickness estimation was performed with the Freesurfer image analysis suite (Dale et al., 1999; Fischl et al., 2000): more detailed methods are available in supplementary online information. To reduce the standard deviation of the thickness measurement across the cohort, we applied a small surface-based Gaussian smoothing of 20 mm full width at half-maximum (selected based on likely effect size) (Dale et al., 1999). A vertex-by-vertex linear regression analysis was performed using the SurfStat software (http://www.stat.uchicago.edu/∼worsley/surfstat/) to examine differences in cortical thickness between the patient groups and the control group. Cortical thickness, C, was modeled as a function of group, controlling for age, gender and total intracranial volume by including them as nuisance covariates. C = β1 SD + β_2_ PNFA + β_3_ LPA + β_4_ controls + β_5_ age + β_6_ gender + β_7_ TIV + μ + ɛ (where μ is a constant, and ɛ is error). In separate analyses, we compared subgroups with AD-PPA (the five patients with CSF biomarker data consistent with Alzheimer pathology) and *GRN-*PPA (the two patients with *GRN* mutations) with each other and with SD, PNFA, and controls. Contrasts of interest between the estimates of the group parameters were assessed using two-tailed t-tests. Maps showing statistically significant differences between each disease group and healthy controls were generated and corrected for multiple comparisons to control the False Discovery Rate (FDR) at a 0.001 significance level. For disease group comparisons maps were thresholded at an FDR corrected 0.05 significance level.

#### Voxel-based morphometry analysis

Voxel-based morphometry (VBM) was performed using SPM5 software (http://www.fil.ion.ucl.ac.uk/spm) and the DARTEL toolbox with default settings for all parameters: more detailed methods are available in supplementary online information. Linear regression models were used to examine differences in GM and WM volume between the groups. Voxel intensity, V, was modeled as a function of group, and subject age gender and total intracranial volume were included as nuisance covariates. V = β_1_ SD + β_2_ PNFA + β_3_ LPA + β_4_ controls + β_5_ age+ β_6_ gender + β_7_ TIV + μ + ɛ (where μ is a constant, and ɛ is error). Similar analyses were performed separately for the AD-PPA and *GRN*-PPA subgroups. The analysis was performed over voxels inside a “consensus mask” (Ridgway et al., 2009), which included all voxels where intensity > 0.1 was present in > 70% of subjects. Separate analyses were performed on the grey and white matter segments. Maps showing single-tailed statistically significant differences between the groups were generated, correcting for multiple comparisons in the disease group-control comparisons by thresholding the images of t-statistics to control the False Discovery Rate (FDR) at a 0.05 significance level. For disease group comparisons maps were generated uncorrected at a 0.001 significance level. Statistical parametric maps were displayed as overlays on a study-specific template, created by warping all native space whole-brain images to the final DARTEL template and calculating the average of the warped brain images.

## Results

### Volumetric analysis

All PPA groups had asymmetrical predominantly left-sided cerebral atrophy (Table 2). However, hemispheric asymmetry was more marked in the SD and combined LPA groups. The subgroup analysis revealed markedly asymmetric atrophy in the *GRN*-PPA subgroup (L/R ratio = 0.83) which was significantly more asymmetric than all other disease groups; atrophy in the AD-PPA subgroup was similar to the SD group in terms of asymmetry (L/R ratio = 0.94) and significantly more asymmetric than the PNFA group.

Subcortical volumetric data showed smaller caudate volumes bilaterally in the PNFA group compared to controls (with a trend to smaller brainstem volume also) and smaller left hippocampal and bilateral amygdala volumes in the SD group compared to controls. In the combined LPA group, the left caudate, hippocampus and amygdala were significantly smaller than controls and these findings were similar in the AD-PPA subgroup, while the left hippocampus was significantly smaller only in the *GRN*-PPA subgroup.

### Cortical thickness analysis

**Fig. 1:**
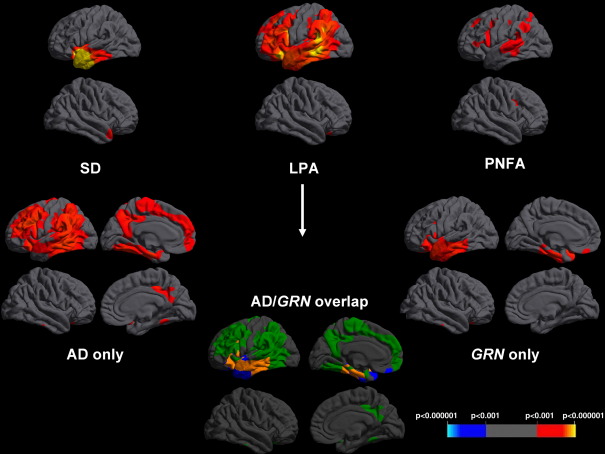
Cortical thickness maps showing patterns of cortical thinning in disease groups compared to healthy controls. For each disease panel, left hemisphere sections are shown above and right hemisphere sections below. Maps are thresholded at *p* < 0.001 after FDR correction over the whole brain volume. The colored bar represents FDR corrected p-values. Within the LPA group, the composite map “AD/GRN overlap” codes areas in which cortical thinning was observed only in the AD-PPA group (green), only in the GRN-PPA group (blue) or in both groups (orange) relative to healthy controls at the prescribed threshold.

**Fig. 2:**
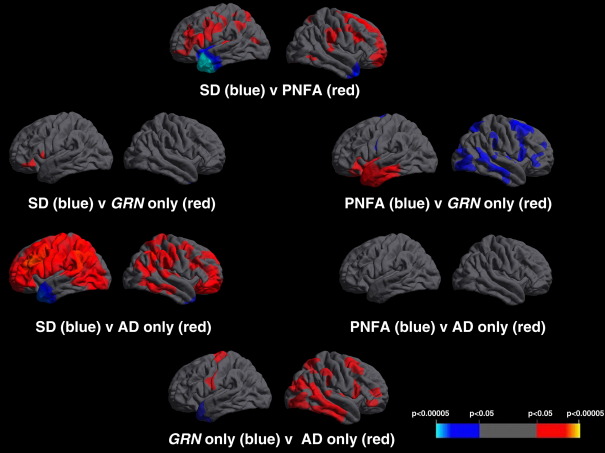
Cortical thickness maps showing patterns of cortical thinning in between disease-group differences. For each disease panel, left hemisphere sections are shown on the left and right hemisphere sections on the right. Maps are thresholded at *p* < 0.05 after FDR correction over the whole brain volume. The colored bar represents FDR corrected *p*-values.

Compared with healthy controls, cortical thinning was predominantly left-sided in all three PPA groups (Fig. 1, Table 2B). The SD group showed involvement of the antero-inferior temporal lobes (left greater than right and particularly the temporal pole, parahippocampal and entorhinal cortex) and to a lesser extent the left frontal lobe (particularly orbitofrontal cortex) (Fig. 1) while in the PNFA group there was maximal involvement of the left inferior frontal (pars triangularis and pars opercularis), superior frontal, insular and superior temporal cortex with lesser involvement of the anterior parietal lobe (Fig. 1). In the combined LPA group there was more widespread involvement of the left hemisphere with the areas of most significant cortical thinning in the posterior temporal lobe (particularly superior and middle temporal gyri), medial temporal lobe, inferior parietal lobe and frontal lobe (inferior, middle and orbitofrontal gyri) with lesser involvement of the precuneus (Fig. 1). The subgroup analysis of *GRN*-PPA and AD-PPA revealed overlapping but distinct patterns in the two subgroups compared to controls (Fig. 1): both groups had mid to posterior temporal lobe and inferior frontal involvement but in the AD-PPA group there was greater temporo-parietal junction and frontal atrophy and in the *GRN*-PPA group there was more anterior temporal atrophy (Fig. 1). Comparing patterns of maximal cortical thinning between the AD-PPA and *GRN-*PPA subgroups and the other disease groups, there was greater posterior temporal, inferior parietal and inferior frontal lobe involvement in the AD-PPA subgroup than the SD group (with more anterior temporal lobe involvement in SD), but no significant differences with respect to the PNFA group; while in the *GRN*-PPA subgroup there was greater left inferior frontal lobe involvement than the SD group (with more right anterior temporal lobe thinning in SD), and more anterior temporal lobe involvement compared to the PNFA group (with more right hemisphere involvement in PNFA) (Fig. 2). On direct comparison of the LPA subgroups, the AD-PPA subgroup had more marked thinning of left anterior parietal cortex and extensive cortical areas in the right cerebral hemisphere, while the *GRN*-PPA subgroup had more marked thinning of left anterior temporal cortex (Fig. 2).

### VBM analysis

**Fig. 3:**
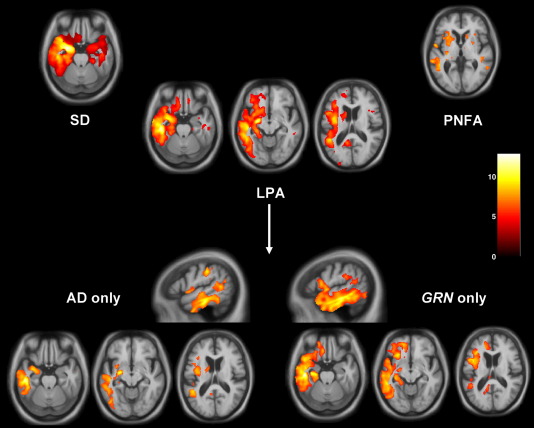
VBM analysis on grey matter regions in PPA groups relative to healthy controls. For each axial section, the left hemisphere is shown on the left; sagittal sections are through the left hemisphere. Maps are thresholded at *p* < 0.05 after FDR correction over the whole brain volume. Grey matter differences are color coded (red-yellow) in terms of t-score as indicated on the color bar (right).

**Fig. 4:**
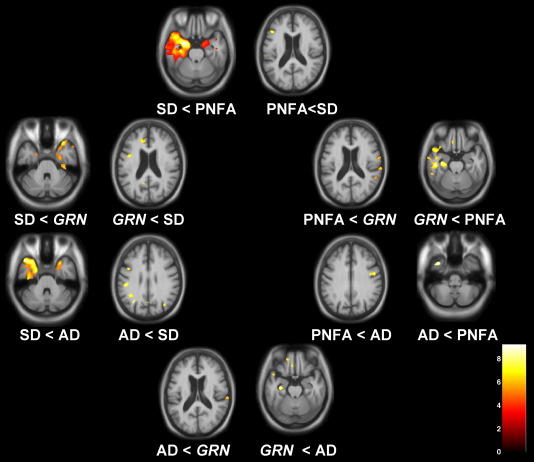
VBM analysis on grey matter regions in disease group comparisons. For each axial section, the left hemisphere is shown on the left. Maps are thresholded at *p* < 0.001 uncorrected. Grey matter differences are color coded (red-yellow) in terms of *t*-score as indicated on the color bar (lower right).

The VBM analysis corroborated the findings of the cortical thickness analysis with similar findings in the SD and PNFA groups compared to controls and in the LPA group, maximal involvement of the left posterior temporal, inferior parietal and inferior frontal lobes compared to controls (Fig. 3). In the subgroup analyses in relation to healthy controls, patterns of grey matter atrophy overlapped in the AD-PPA and *GRN*-PPA subgroups, but the AD-PPA subgroup had greater posterior (particularly parietal) involvement while the *GRN*-PPA subgroup had greater anterior temporal lobe involvement (Fig. 3). The findings differed from the cortical thickness measures in showing greater overlap between the AD-PPA and *GRN*-PPA subgroups in posterior temporal, inferior parietal and inferior frontal lobe areas. Comparing patterns of most significant grey matter loss in the LPA subgroups, the AD-PPA subgroup had greater left posterior temporal, parietal and inferior frontal atrophy than the SD group, and greater left inferior temporal involvement than the PNFA group; while the *GRN*-PPA subgroup had greater inferior frontal and precuneus involvement than the SD group, and greater temporal lobe involvement than the PNFA group (Fig. 4). On direct comparison of the LPA subgroups, the AD-PPA subgroup had greater atrophy in biparietal and right posterior temporal cortices, while the *GRN*-PPA subgroup had greater atrophy of left anterior and inferior temporal and left orbitofrontal cortex (Fig. 4).

**Fig. 5:**
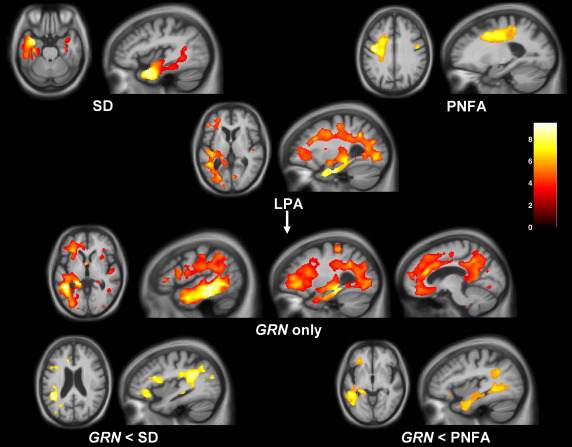
VBM analysis on white matter regions in PPA subgroups relative to healthy controls and (bottom row) in the *GRN*-PPA subgroup relative to other disease groups. For each axial section, the left hemisphere is shown on the left; sagittal sections are through the left hemisphere. For control comparisons, maps are thresholded at *p* < 0.05 after FDR correction over the whole brain volume; for disease group comparisons, maps are thresholded at *p* < 0.001 uncorrected. White matter differences are color coded (red-yellow) in terms of t-score as indicated on the color bar (right).The AD-PPA subgroup showed no significant areas of white matter loss relative to other disease groups at the prescribed threshold.

The white matter analysis revealed distinct patterns of tract involvement in each of the three groups: in the SD group, there was involvement of white matter tracts predominantly in the left temporal lobe including the fornix, inferior longitudinal fasciculus and uncinate fasciculus (Fig. 5); in the PNFA group, there was maximal involvement of a left frontal lobe white matter region likely to represent part of the superior longitudinal fasciculus (Fig. 5); and in the LPA group there was more widespread white matter involvement predominantly in the left hemisphere, including the intrahemispheric long association tracts (inferior longitudinal fasciculus, superior longitudinal fasciculus, inferior fronto-occipital fasciculus and cingulum) as well as the fornix (Fig. 5). Comparing patterns of maximal white matter loss between disease groups relative to healthy controls (Fig. 5), the *GRN*-PPA subgroup had most marked involvement of intrahemispheric long association tracts including inferior longitudinal fasciculus, superior longitudinal fasciculus, inferior fronto-occipital fasciculus and cingulum, and also involvement of the corpus callosum and brainstem tracts. The *GRN*-PPA subgroup showed greater involvement of dorsal fronto-parietal tracts than the SD group, greater involvement of temporal lobe tracts than the PNFA group, and greater involvement of both fronto-parietal and temporal lobe tracts than the AD-PPA subgroup. The AD-PPA subgroup had no significant white matter involvement relative to either healthy controls or the disease subgroups.

## Discussion

Here we present a detailed neuroanatomical characterization of LPA in comparison to the canonical PPA subtypes, SD and PNFA; and in particular, neuroanatomical signatures of LPA subgroups with *GRN* mutations and with probable AD pathology. Allowing for the different modalities used and the limited spatial resolution of smoothed data which preclude fine-grained anatomical correlation, complementary cortical thickness and morphometric techniques here have shown a broadly convergent pattern of findings. The LPA syndrome is associated with asymmetrical atrophy predominantly of the left hemisphere with particular involvement of more posterior cortical areas (including posterior superior temporal/inferior parietal areas and precuneus) that chiefly discriminates this syndrome anatomically from other subtypes of PPA. The white matter VBM analysis here reveals that this profile of cortical damage is underpinned by involvement chiefly of long association tracts in the left hemisphere. Although there is overlap between the LPA subgroup with AD pathology and the subgroup with *GRN* mutations, there are distinct patterns of atrophy with more posterior temporo-parietal junction and frontal lobe involvement in AD-PPA and more anterior temporal lobe involvement in *GRN*-PPA. These neuroanatomical findings are consistent with differences in the neuropsychological profiles of these two groups. Cortical atrophy in the AD-PPA group here appears more extensive than previously reported in LPA (Gorno-Tempini et al., 2004, 2008): this may have been a correlate of relatively more severe disease in our AD-PPA group. This severity issue might also account for the more extensive intrahemispheric atrophy of the LPA cases in relation to the SD and PNFA cases here. However, interpretation of severity effects is problematic where severity measures are closely correlated with the specific effects of the disease process: clues that severity is not the entire explanation for the extensive left hemispheric damage in our LPA group are the somewhat shorter mean disease duration for the combined LPA group (Table 1) and the striking asymmetry of both anatomical damage and neuropsychological functions (e.g. normal object perception: Table 1) in the *GRN*-PPA subgroup, suggesting that the disease process in these cases preferentially affects a distributed left hemisphere network.

Our findings in the SD and PNFA groups corroborate the work of previous studies, and provide further information about the integrity of white matter pathways that are likely to be critical in binding cortical areas into distributed networks that mediate particular language functions (Scott et al., 2003; Spitsyna et al., 2006; Awad et al., 2007; Seeley et al., 2009). In SD there was asymmetrical, left greater than right anterior temporal lobe atrophy with less marked involvement of orbitofrontal cortex (Galton et al., 2001; Rosen et al., 2002; Rohrer et al., 2009). Of note, all of the SD cases here had left temporal lobe onset (no cases with right temporal lobe onset were ascertained during the period of the study). In PNFA there was left inferior frontal lobe, insula and superior temporal lobe atrophy with less marked involvement of the caudate and anterior parietal lobe (Nestor et al., 2003; Gorno-Tempini et al., 2004; Ogar et al., 2007; Rohrer et al., 2009). White matter disease has been little studied in SD and PNFA, however one diffusion tensor imaging study in a mixed “temporal variant” FTLD cohort (Borroni et al., 2007) showed involvement of white matter tracts, including inferior longitudinal fasciculus, inferior fronto-occipital fasciculus, callosal and superior longitudinal fasciculus. The present study with stratification of PPA subgroups is consistent both with previous neuroanatomical findings and with the distinctive neuropsychological profiles of SD and PNFA. In SD, there was predominant involvement of anterior temporal cortices and white matter tracts (fornix, inferior longitudinal fasciculus and uncinate fasciculus) implicated in semantic processing (Spitsyna et al., 2006); while in PNFA, there was predominant involvement of inferior frontal, insular and parieto-temporal cortices and dorsal white matter tracts (including the superior longitudinal fasciculus) implicated in speech production (Scott et al., 2003).

The LPA syndrome is defined by the presence of a primary language disorder with the key constellation of impoverished though non-effortful spontaneous speech marred by prominent word-finding pauses and less prominent phonemic errors, anomia, impaired sentence comprehension and impaired repetition particularly of sentences (Gorno-Tempini et al., 2004, 2008). This language disorder is associated with reduced digit span (indicative of a phonological store deficit). Neuropsychological assessment of the present cases corroborated these features and demonstrated additional dominant parietal lobe deficits (dyscalculia, deep/phonological dyslexia or limb apraxia, alone or in combination). Although a primary defect of phonological working memory has been proposed in LPA (Gorno-Tempini et al., 2008), it is unlikely that the primary cognitive defect in this degenerative syndrome is restricted to a single information processing module. For example, anomia and word-finding pauses might reflect a primary word retrieval deficit or a more specific phonological access deficit linked to disruption of inferior parietal or posterior superior temporal lobe areas, while limb apraxia is likely to reflect involvement of a distinct network mediating the control of voluntary action that includes the left parietal lobe. The pattern of deficits in LPA suggests involvement of the left parieto-temporal junction and retrosplenial region and functional connections in the dorsal language processing stream linking to inferior frontal areas (Rohrer et al., 2008a; Awad et al., 2007; Wong et al., 2009). This pattern is likely to be relatively specific for LPA: a similar pattern has emerged in previous neuroanatomical studies of the syndrome (Gorno-Tempini et al., 2004, 2008), and furthermore, direct comparison with SD and PNFA cases here revealed distinct group-specific patterns of atrophy involving both cortex and white matter tracts. We propose that LPA is a network-based syndrome that implicates distributed dominant hemisphere cortices and white matter connections previously shown to be critical for the production and analysis of language in normal functional imaging (Awad et al., 2007) and focal lesion (Hillis, 2007) studies.

The finding that the majority of the LPA cases had CSF biomarkers in keeping with AD pathology is consistent with a previous study in which 64% (7/11 cases) of patients with LPA had AD pathology: most of the other cases had FTLD-U pathology, though further genetic analysis was not undertaken (Mesulam et al., 2008). If LPA signals an atypical language presentation of AD in a high proportion of cases, it is noteworthy that the pattern of anatomical changes we have delineated here could be interpreted as a highly asymmetrical variant of the anatomical profile described in typical amnestic AD, with involvement of the medial temporal lobe, temporo-parietal junction and precuneus (Scahill et al., 2002). Indeed, language dysfunction and parietal signs frequently develop in the course of typical amnestic AD (Croot et al., 2000; Harasty et al., 2001; Taler et al., 2008) and an atypical language variant of AD has been described, of which many cases appear to have had an LPA syndrome (Galton et al., 2000; Alladi et al., 2007).

In this study, 22% of patients diagnosed with LPA (2/9) were found to have *GRN* mutations, which have been shown previously to be associated with asymmetrical hemispheric cortical atrophy frequently involving the parietal lobe (Beck et al., 2008; Rohrer et al., 2008b; Le Ber et al., 2008). The *GRN*-PPA group fitted most closely the LPA subtype of PPA rather than the other two subtypes (and were so classified prior to discovery of their mutation), and it is noteworthy that no *GRN* mutations were discovered in patients representing other PPA syndromes in this series. However, detailed subgroup analysis of neuroimaging and neuropsychological data suggests that despite the overlap there are certain features which may help to distinguish LPA in association with *GRN* mutations from LPA likely to be caused by AD pathology. On neuropsychological assessment, our *GRN*-PPA patients had more severe deficits of naming, single word comprehension, and vocabulary-based (irregular word) reading. Anatomically (in common with SD), this argues for involvement of dominant anterior temporal lobe mechanisms in *GRN*-PPA, and indeed, the neuroimaging signature of *GRN*-PPA here was strikingly asymmetric, with more severe anterior temporal lobe involvement (and more severe white matter involvement) than with AD-PPA. This neuroanatomical correlate implicates the ventral language processing pathway linking the posterior superior temporal lobe with more anterior temporal areas in the dominant hemisphere (Spitsyna et al., 2006), suggesting that the *GRN*-associated subtype of LPA may be a dual-pathway disease. Caution is clearly required in interpreting these findings, due both to the small number of patients studied and the current lack of pathological confirmation in the non-*GRN* LPA group: further work is needed to characterize the *GRN*-PPA syndrome fully and to establish its true relation to non-*GRN* LPA, as well as PNFA and SD. We regard the present data as important preliminary evidence which will require substantiation in future studies with larger cohorts and histopathological correlation.

In summary, LPA has a neuroanatomical profile that is consistent with the clinical and neuropsychological features of this syndrome. The LPA profile overlaps with other PPA syndromes, but is distinguished chiefly by more extensive involvement of posterior elements of the language network. AD pathology is likely to account for most cases of LPA, with a significant minority of other molecular pathologies, notably *GRN* mutations, causing a similar syndrome. As with any disorder producing aphasia, LPA provides information about the organization of language networks that is complementary to functional imaging studies in healthy subjects, by delineating areas that are critical for (rather than simply associated with) particular functions. However, the specific neurobiological importance of our findings is threefold. Firstly, the LPA syndrome as a clinico-anatomical entity has a profile of brain damage that is complementary to the previously described “network-based” disorders of SD and PNFA. Secondly, LPA suggests a mechanism by which involvement of dominant temporo-parietal junctional areas produces a core deficit affecting manipulation of phonological information with spread of disease through anatomically and functionally connected language pathways: this predicts a pattern of disease evolution in LPA which could be tested empirically in longitudinal studies. Finally, the association of a similar syndrome with *GRN* mutations provides a specific molecular substrate for language network dysfunction. Viewed from this perspective, LPA represents a unique “experiment of nature” that illustrates the effects of progressive erosion of the distributed human language network. Further behavioral and pathophysiological studies including detailed cross-sectional and longitudinal correlation of neurolinguistic functions with anatomical substrates are needed to define the core components of the LPA syndrome and their brain basis, which may lie ultimately with disordered connectivity in distributed dominant hemispheric networks.

## Figures and Tables

**Table 1:** Demographic and neuropsychological data.

Mean (standard deviation)	SD	PNFA	LPA combined	GRN only	AD only	Controls
Number of subjects	9	14	9	2	5	18
%Male	33.3	71.4	55.6	50.0	80.0	50.0
Age (years)	62.3 (9.0)	71.8 (6.8)	64.1 (7.4)	60.7 (12.7)	63.1 (4.4)	67.9 (5.4)
Duration from symptom onset (years)	5.3 (1.2)	5.3 (2.1)	4.2 (0.9)	3.7 (0.0)	4.5 (1.0)	N/A
Mini-Mental State Examination (/30)	22.7 (5.2)^a^	24.4 (5.6)^a^	15.9 (5.2)^a,e,f^	16.0 (2.8)^a^	13.8 (5.7)^a^	29.7 (0.8)
Range of scores	14–28	12–30	8–22	14–18	8–22	27–30
CDR score	0.6 (0.2)^a^	0.6 (0.3)^a^	0.8 (0.3)^a^	0.8 (0.4)^a^	0.7 (0.3)^a^	0.0 (0.0)
Range of scores	0.5–1	0–1	0.5–1	0.5–1	0.5–1	0
CDR sum of boxes	2.4 (1.5)^a^	2.4 (1.3)^a^	4.5 (1.4)^a,e,f^	4.0 (2.8)^a^	4.4 (1.3)^a^	0.0 (0.0)
Range of scores	(0.5–5.5)	0–4.5	2–6	2–6	3–5.5	0
Frontal Assessment Battery⁎ (/18)	14.2 (2.2)^a^	11.4 (3.8)^a,d^	8.1 (2.0)^a,e,f^	*9.5 (0.7)*^*a*^	*7.4 (1.8)*^*a*^	17.8 (0.4)

*Language*						
Naming task (/20)	4.4 (3.2)^a,b^	12.7 (6.4)^a^	4.0 (4.3)^a,f^	0.0 (0.0)^a^	5.6 (4.6)^a^	19.7 (0.7)
Single-word repetition task (/30)	29.7 (1.0)	23.4 (9.7)^a,d^	22.3 (9.7)^a,e^	18.5 (2.1)^a^	26.6 (5.3)	29.8 (0.4)
Sentence repetition task (/10)	NT^1^	6.3 (4.1)^a^	3.7 (4.0)^a,f^	0.5 (0.7)^a^	5.0 (4.3)^a^	10.0 (0.0)
British Picture Vocabulary Scale (/30)	15.1 (5.2)^a,b,c^	25.4 (4.0)^a^	19.9 (3.5)^a,f^	15.5 (2.1)^a^	20.0 (2.3)^a^	28.3 (0.9)
Test for reception of grammar (/20)	16.3 (2.6)^a^	15.5 (3.1)^a^	12.0 (3.5)^a,e,f^	10.5 (0.7)^a^	12.6 (4.8)^a^	19.0 (0.9)
Irregular word reading task (/30)	15.4 (8.3)^a^	18.1 (8.6)^a^	11.4 (6.9)^a,f^	4.5 (0.7)^a^	14.4 (6.6)^a^	28.3 (1.7)
Graded nonword reading test (/20)	NT^1^	9.6 (6.6)^a^	7.7 (6.9)^a^	6.0 (8.5)^a^	11.3 (5.5)^a^	19.7 (0.7)

*Memory*						
Camden Pictorial Recognition Memory Test (/30)	28.2 (2.2)	29.4(0.8)	24.8 (5.1)^a,e,f^	23.0 (9.9)^a^	24.2 (4.5)^a^	29.7 (0.8)

*Executive function*						
Trail making test A (scaled score)	8.1 (2.8)	4.1 (2.2)^a,d^	4.7 (3.1)^a,e^	4.0 (0.0)^a^	4.5 (3.9)^a^	9.5 (2.8)
Trail making test B (scaled score)	8.0 (3.3)	4.5 (3.3)^a,d^	2.5 (1.1)^a,e^	2.4 (0.2)^a^	2.2 (0.8)^a^	9.9 (2.5)

*Other cognitive domains*						
Digit span forwards^2^	6.8 (1.5)	5.1 (1.4)^a,d^	3.7 (1.7)^a,e,f^	2.5 (0.7)^a^	3.8 (2.2)^a^	6.9 (0.6)
Object decision (VOSP) (/20)	17.2 (2.8)	16.7 (2.3)	16.7 (2.4)	19.0 (0.0)	15.6 (2.8)	17.5 (2.3)
Graded Difficulty Arithmetic Test (/12)^3^	5.5 (4.2)	3.0 (3.7)^a^	1.1 (1.8)^a,e^	1.5 (2.1)^a^	0.0 (0.0)^a^	7.2 (2.1)
Limb apraxia (% of cases)	0.0	50.0	100.0	100.0	100.0	0.0

Statistically significant differences between the SD, PNFA, LPA and control groups are represented by superscript letters: ^a^*p* < 0.05 disease group significantly worse than controls, ^b^*p* < 0.05 SD worse than PNFA, ^c^*p* < 0.05 SD worse than LPA, ^d^*p* < 0.05 PNFA worse than SD, ^e^*p* < 0.05 LPA worse than SD, ^f^*p* < 0.05 LPA worse than PNFA. For the LPA subgroups of *GRN* only and AD only a superscript letter^a^ represents *p* < 0.05 disease group significantly worse than controls. ^1^SD patients not tested (NT) on sentence repetition task or Graded Nonword Reading Test, ^2^5 SD and all of the PNFA, LPA and controls performed this test, ^3^4 SD, 5 PNFA, all of the LPA and 6 controls performed this test.

⁎ The Frontal Assessment Battery (Dubois et al., 2000) comprises the following subtests: conceptualization, mental flexibility, motor programming, sensitivity to interference, inhibitory control, and environmental autonomy.

**Table 2:** (A) Volumetric data for whole brain, left and right cerebral hemisphere, caudate, hippocampus and amygdala volumes as a percentage of total intracranial volume (TIV), (B) cortical thickness data for the frontal, temporal and parietal lobes.

A						
Cerebral region volumes (as a percentage of TIV), mean (standard deviation)	SD	PNFA	LPA	GRN only	AD only	Controls
Whole brain	68.1 (3.8)	64.2 (5.7)^a,d^	65.1 (5.6)^a^	63.2 (0.8)	66.3 (6.3)	70.1 (4.0)
Left hemisphere	32.7 (2.0)	31.1 (2.9)^a^	30.7 (3.0)^a^	28.3 (0.2)^a^	31.8 (2.9)^a^	34.4 (1.9)
Right hemisphere	34.7 (1.8)	32.2 (2.8)^a,d^	33.6 (2.8)	33.9 (0.3)	33.7 (3.4)	34.6 (2.0)
Left/right hemispheric ratio	0.94 (0.01)^a,b^	0.97 (0.04)^a^	0.91 (0.05)^a,f^	0.83 (0.00)^a^	0.94 (0.01)^a^	1.00 (0.01)
Brainstem	1.26 (0.11)	1.20 (0.15)	1.22 (0.12)	1.21 (0.07)	1.23 (0.14)	1.27 (0.10)
Left caudate	0.20 (0.03)	0.19 (0.03)^a^	0.19 (0.02)^a^	0.19 (0.01)	0.18 (0.01)^a^	0.22 (0.03)
Right caudate	0.22 (0.03)	0.20 (0.02)^a^	0.22 (0.03)	0.24 (0.01)	0.20 (0.02)	0.23 (0.04)
Left hippocampus	0.13 (0.04)^a,b,c^	0.20 (0.03)	0.18 (0.05)^a^	0.15 (0.02)^a^	0.19 (0.04)^a^	0.22 (0.03)
Right hippocampus	0.21 (0.03)	0.22 (0.03)	0.22 (0.04)	0.21 (0.00)	0.22 (0.04)	0.24 (0.03)
Left amygdala	0.04 (0.02)^a,b,c^	0.08 (0.01)	0.07 (0.02)^a^	0.07 (0.02)	0.07 (0.02)^a^	0.08 (0.01)
Right amygdala	0.07 (0.02)^a,b,c^	0.08 (0.01)	0.09 (0.02)	0.11 (0.00)	0.09 (0.02)	0.09 (0.01)


*B*							
Cortical thickness in each lobe (mm), mean (standard deviation)							
Frontal	Left	2.2 (0.1)	2.0 (0.2)^a,d^	1.9 (0.1)^a,e^	1.9 (0.1)^a^	1.9 (0.1)^a^	2.2 (0.1)
Right	2.3 (0.1)	2.1 (0.1)^a,d^	2.1 (0.1)^e^	2.3 (0.0)	2.1 (0.1)^a^	2.2 (0.1)
Temporal	Left	1.7 (0.2) ^a,b^	2.1 (0.3)^a^	1.8 (0.1)^a,f^	1.6 (0.4)^a^	1.9 (0.1)^a^	2.4 (0.1)
Right	2.2 (0.2)^a^	2.2 (0.2)^a^	2.3 (0.1)	2.5 (0.1)	2.2 (0.1)^a^	2.4 (0.1)
Parietal	Left	2.0 (0.1)	1.8 (0.2)^a,d^	1.7 (0.1)^a,e^	1.8 (0.1)^a^	1.7 (0.1)^a^	2.0 (0.1)
Right	2.1 (0.1)	1.9 (0.2)^a,d^	2.0 (0.1)^e^	2.2 (0.1)	1.8 (0.1)^a^	2.0 (0.1)

Statistically significant differences between the SD, PNFA, LPA and control groups are represented by superscript letters: ^a^*p* < 0.05 disease group significantly worse than controls, ^b^*p* < 0.05 SD worse than PNFA, ^c^*p* < 0.05 SD worse than LPA, ^d^*p* < 0.05 PNFA worse than SD, ^e^*p* < 0.05 LPA worse than SD, ^f^*p* < 0.05 LPA worse than PNFA. For the LPA subgroups of *GRN* only and AD only a superscript letter^a^ represents *p* < 0.05 disease group significantly worse than controls.
